# Risk factors for osteoporotic hip fracture among community-dwelling older adults: a real-world evidence study

**DOI:** 10.1186/s42358-024-00350-6

**Published:** 2024-01-17

**Authors:** Daniela Castelo Azevedo, Leonardo Santos Hoff, Sergio Candido Kowalski, Carlos Augusto Ferreira de Andrade, Virgínia Fernandes Moça Trevisani, Ana Karla Guedes de Melo

**Affiliations:** 1 Data Science and Epidemiology, Clínica Mais 60 Saúde, Rua Juiz de Fora, 1071 - Barro Preto, Belo Horizonte, MG 30180-060 Brazil; 2 School of Medicine, Universidade Potiguar (UnP), Natal, RN Brazil; 3 Department of Health Research Methods, Evidence, and Impact (HEI), Faculty of Health Sciences, McMaster University, Hamilton, ON Canada; 4 Department of Medical Clinic, Universidade Federal do Paraná, Curitiba, Brazil; 5 Department of Epidemiology and Quantitative Methods in Health, National School of Public Health Sergio Arouca (ENSP)/ Oswaldo Cruz Foundation (FIOCRUZ), Rio de Janeiro, RJ Brazil; 6 Faculty of Medicine, Vassouras University, Rio de Janeiro, RJ Brazil; 7 Department of Emergency Medicine and Evidence Based Health Program, Universidade Federal de São Paulo, São Paulo, SP Brazil; 8 Department of Rheumatology, Universidade de Santo Amaro, São Paulo, SP Brazil; 9 Department of Rheumatology, Hospital Universitário Lauro Wanderley/Universidade Federal da Paraíba, João Pessoa, PB Brazil; 10 Evidence Based Health Program, Universidade Federal de São Paulo, São Paulo, SP Brazil

**Keywords:** Hip fractures, Community-dwelling older adults, Patient-reported outcomes

## Abstract

**Background:**

Hip fractures in the older adults lead to increased morbidity and mortality. Although a low bone mineral density is considered the leading risk factor, it is essential to recognize other factors that could affect the risk of hip fractures. This study aims to evaluate the contribution of clinical characteristics, patient-reported outcomes, and muscle and aerobic capacity for hip fractures in community-dwelling older adults.

**Methods:**

This is a retrospective cohort study with real world-data from subjects ≥ 60 years old attending an outpatient clinic in Minas Gerais, Brazil, from May 1, 2019, to August 22, 2022. Data about clinical characteristics (multimorbidity, medications of long-term use, sedative and or tricyclic medications, number of falls), patient-reported outcomes (self-perception of health, self-report of difficulty walking, self-report of vision problems, and self-report of falls) and muscle and aerobic capacity (calf circumference, body mass index, and gait speed) were retrieved from an electronic health record. The association of each potential risk factor and hip fracture was investigated by a multivariable logistic regression analysis adjusted for age and sex.

**Results:**

A total of 7,836 older adults were included with a median age of 80 years (IQR 72–86) and 5,702 (72.7%) were female. Hip fractures occurred in 121 (1.54%) patients. Multimorbidity was associated with an increased risk of hip fracture (OR = 1.12, 95%CI 1.06–1.18) and each episode of fall increased the chance of hip fracture by 1.7-fold (OR = 1.69, 95%CI 1.52–1.80). Patient-reported outcomes associated with increased fracture risk were regular or poor self-perception of health (OR = 1.59, 95%CI 1.06–2.37), self-report of walking difficulty (OR = 3.06, 95%CI 1.93–4.84), and self-report of falls (OR = 2.23, 95%CI 1.47–3.40). Body mass index and calf circumference were inversely associated with hip fractures (OR = 0.91, 95%CI 0.87–0.96 and OR = 0.93, 95%CI 0.88–0.97, respectively), while slow gait speed increased the chance of hip fractures by almost two-fold (OR = 1.80, 95%CI 1.22–2.66).

**Conclusion:**

Our study reinforces the importance of identified risk factors for hip fracture in community-dwelling older adults beyond bone mineral density and available fracture risk assessment tools. Data obtained in primary care can help physicians, other health professionals, and public health policies to identify patients at increased risk of hip fractures.

## Background

Hip fractures are associated with increased disability, morbidity, and mortality [1–3], and their incidence has been increasing worldwide concurrently with population aging [3]. They usually occur in the eighth decade of life, mainly among women, with a lifetime risk of 14% in women and 3% in men [4]. The 1-year mortality after the fracture is 22–29% [1], and many survivors have a permanent disability, being no longer able to walk and requiring long-term nursing home care [4]. Therefore, hip fractures in older adults represent a major global health issue.

Most fractures in this age group occur in patients with osteoporosis due to low-energy trauma and are therefore defined as fragility fractures [5]. The fracture risk is usually assessed by measuring the bone mineral density using dual-energy X-ray absorptiometry (DXA). However, this exam has limitations and may only be available in some settings [6]. Other factors have been shown to independently contribute to fracture risk, like age, previous fragility fractures, tobacco and alcohol use, glucocorticoids, chronic inflammatory diseases, and propensity to falls [7, 8]. Tools to improve fracture risk prediction by combining DXA and clinical information were developed and are widely used in clinical practice [7, 9].

Patient-reported outcomes (PROs) provide relevant information about the patient’s self-perception of many domains of health [10]. They are easily obtained and can be compared among different populations and medical conditions [10]. The Clinical-Functional Vulnerability Index-20 (IVCF-20) is a PRO measure with a good correlation with other comprehensive geriatric assessment tools. Brazilian primary care physicians and geriatricians used the IVCF-20 to identify older adults at risk of frailty [11]. Some PROs reported in the IVCF-20, like the overall perception of health, walking difficulty, vision problems, and falls may be related to an increased risk of hip fracture, although this relationship has not been studied. Body mass index (BMI), calf circumference, and gait speed test are also assessed in the IVCF-20 and could help to identify older people at risk of hip fractures.

This study aims to evaluate if clinical characteristics, PROs related to frailty, and muscle and aerobic capacity are associated with the risk of hip fractures in community-dwelling older adults.

## Methods

### Population

A retrospective cohort study was conducted using real-world data from a digital health care platform (LifeCode, EFG Inteligencia e Consultoria Ltda, Belo Horizonte, Brazil). Real-world data is information relating to patient’s health status or the delivery of health care derived from sources other than typical clinical research settings [12]. The trained health professionals have used this platform to register all clinical visits and telehealth of community-dwelling older adults attending a geriatric outpatient clinic in the Southeastern Brazilian city of Belo Horizonte from May 1, 2019, to August 22, 2022. The platform has a user-friendly electronic health record (EHR) with structured entry forms for clinical information, e.g., diagnosis, medications in use, presenting symptoms, anthropometric measures, and standardized scales such as IVCF-20, a measure for the assessment of frailty in patients ≥ 60 years old from the community [11]. It helps with longitudinally patient follow-up by a multidisciplinary team (physicians, nurses, physical therapists, psychologists, nutritionists, speech therapists, and pharmacists), aiming to preserve functionality and avoid adverse outcomes, such as falls, unnecessary hospitalizations, and polypharmacy.

We included all patients with valid data in the study period.

### Assessment of hip fracture

The record of any hip fracture hospitalization in the EHR during the period of study was considered an osteoporotic hip fracture. This definition of hip fracture has usually been used in epidemiological studies using databases [13]. The nursing team confirms every hospitalization reported by the patient’s health insurance by telemonitoring. The nurse registers hospitalizations details, their causes and requests a copy of the discharge summary of all patients, which is attached to the EHR. When in possession of the discharge summary, the nurse checks whether the description corresponds to a hip fracture, that is, a fracture of the proximal femur between the femoral head and 5 cm distal to the lesser trochanter, usually described as a fracture of the femoral neck, pertrochanteric fracture or subtrochanteric fracture, with their corresponding international classification of diseases (ICD-10 S72.0; S72.1 and S72.2). The nursing team receives periodic training to adequately address and record the cause of hospitalization, especially hospitalizations due to hip fractures, as the hip fracture rate is one of the geriatric outpatient clinic’s pay-for-performance criteria. Therefore, it is a reliable information registered in the EHR. Potential risk factors for hip fracture that was readily available in the EHR and recorded at the time of patient ingress to the clinic, such as clinical characteristics, PROs, and muscle and aerobic capacity were assessed and are described in detail below.

### Clinical characteristics

Clinical characteristics investigated are multimorbidity [14, 15], medications of long-term use, sedative and/or tricyclic medications and the number of falls [16, 17]. Multimorbidity, the co-occurrence of multiple diseases, is a readily available measure in primary care [18] and has been used frequently in epidemiologic studies [19]. Here, it was measured through the number of medical diagnoses. The diagnosis count has been commonly used to assess multimorbidity within primary healthcare [18]. The number of medications with long-term use, the number of falls, and the use of sedative and/or tricyclic medications are listed in specific forms of the HER. The falls are updated regularly during telemonitoring of patients. The following medications were classified as sedatives: alprazolam, bromazepam, clonazepam, diazepam, eszopiclone, phenobarbital, flunitrazepam, lorazepam, midazolam, nitrazepam, zolpidem, and zopiclone. The tricyclic medications were amitriptyline, nortriptyline, imipramine, clomipramine, maprotiline, and cyclobenzaprine.

### Patient-reported outcomes

We included patient-reported outcomes (PROs) from IVCF-20, which has a high degree of validity and reliability and covers multidimensional aspects of the adult’s health condition [11]. IVCF-20 has 20 close-ended questions divided into eight sections: age, health self-perception, functional disabilities, cognition, mood, mobility, communication, and multiple comorbidities [11]. Each section has a specific score that composes a maximum amount of 40 points. The higher the value obtained, the higher the risk of the clinical-functional vulnerability of the older adult [11]. In the present study, we considered the following PROs from IVCF-20: self-perception of health, self-report of difficulty walking, and self-report of vision problems and falls.

The regular or poor self-perception of health was identified by the question: “In general, compared to other people from your age, would you say your health is?” With two response options: (1) excellent, very good or good and (2) regular or poor. The walking difficulties, vision problems, and falls were assessed respectively by the positive responses to the questions: “Do you have difficulty walking that prevents you from performing some daily activity?”, “Do you have vision problems that prevent the performance of some daily activity?” and “Did you have two or more falls in the last year?”.

### Muscle and aerobic capacity

Since sarcopenia is associated with hip fractures [20], we assessed muscle and aerobic capacity using the calf circumference, gait speed test, and BMI, surrogate parameters of sarcopenia readily available in clinical practice settings and included in IVCF-20 [21, 22]. In addition, low BMI is associated with low bone mineral density and a low appendicular lean mass and could be potentially related to hip fractures [23].

The median gait speed in our population (7 s) was larger than the cutoff point suggested by the IVCF-20 (5 s). So, we dichotomized the gait speed into normal or slow (> 11 s to cover 4 m), considering the 90th percentile of our sample.

### Statistical analysis

Descriptive analyses used frequencies and percentages (%) for categorical variables. The Shapiro-Wilk test was used to assess the normality of the distribution of continuous variables. The continuous variables with asymmetric distribution were described by medians and interquartile ranges (IQR), and to compare the differences between patients with and without hip fractures, we used the Mann-Whitney U test, as all continuous variables have a non-normal distribution. Further, for the categorical variables, differences were calculated using the chi-squared test.

We further investigated the association of each potential risk factor and hip fracture using a univariate binary logistic regression to calculate odds ratios (OR) with a 95% confidence interval (CI) and a multivariable model adjusted for age and sex. Analyses were performed using STATA (version 14.0, StataCorpLP, College Station, USA), and a two-sided P-value of < 0.05 was considered statistically significant.

## Results

The study included 7,836 older adults with a median age of 80 years (interquartile range 72–86) and 5,702 (72.7%) were female. Hip fractures occurred in 121 (1.54%) patients. Especially in this population of significantly older adults, the hip fractures were due to low-energy trauma. Patients with at least one hip fracture were older, with more multimorbidity and medications of long term-use and had a higher median number of falls. Regarding PROs, patients with hip fractures reported more frequently regular or poor self-perception of health, vision problems, difficulty walking, and falls. Also, BMI and calf circumference were lower in patients who fractured the hip, and slow gait speed was also more frequent in this group. Table 1 demonstrates the complete characteristics of the patients with and without hip fractures and the comparison between groups.

**Table 1:** Comparison of patients with and without hip fracture

Characteristics	With hip fracture (*n* = 121)	Without hip fracture (*n* = 7,715)	p
**Clinical**			
Median age, years (IQR)	87 (83–91)	80 (72–86)	< 0.001
Female sex, n (%)	92 (76.0)	5,610 (72.7)	0.416
Multimorbidity^a^, median (IQR)	7 (5–10)	6 (4–8)	< 0.001
Medications of long-term use, median (IQR)	5 (3–7)	6 (4–8)	0.039
Sedative and/or tricyclic medications^b^, n (%)	26 (21.5)	1,563 (20.3)	0.740
Number of falls, median (IQR)	2 (1–3)	0 (0–1)	< 0.001
**Patient-reported outcomes**			
Regular or poor self-perception of health, n (%)	49 (49.5)	2,737 (39.2)	0.037
Self-report of vision problems, n (%)	24 (24.3)	1,159 (16.6)	0.043
Self-report of difficulty walking, n (%)	71 (71.7)	2,471 (35.4)	< 0.001
Self-reported falls, n (%)	36 (36.4)	1,240 (17.7)	< 0.001
**Muscle and aerobic capacity**			
BMI^c^ (kg/m²), median (IQR)	24.6 (20.9–27.2)	26.2 (23.5–29.6)	< 0.001
Calf circumference, median (IQR)	34 (31–36)	35 (33–37)	< 0.001
Slow gait speed^d^, n (%)	50 (41.3)	1,489 (19.3)	< 0.001

IQR- interquartile range; BMI - body mass index

^a^number of medical diagnoses; ^b^alprazolam, bromazepam, clonazepam, diazepam, eszopiclone, phenobarbital, flunitrazepam, lorazepam, midazolam, nitrazepam, zolpidem, zopiclone, amitriptyline, nortriptyline, imipramine, clomipramine, maprotiline, and/or cyclobenzaprine; ^c^body mass index; ^d^gait speed > 11 s to cover 4 m

Table 2 describes the univariate and multivariable analysis of potential risk factors for hip fractures. Multimorbidity was associated with an increased risk of hip fracture (OR = 1.12, 95% CI 1.06 to 1.18, *p* < 0.001) and each fall increased the chance of hip fracture by 1.7-fold (OR = 1.69, 95% CI 1.52 to 1.80, *p* < 0.001), regardless the age and sex of the patient.

The PROs that were associated with increased fracture risk were regular or poor self-perception of health (OR = 1.59, 95% CI 1.06 to 2.37, *p* = 0.022), walking difficulty (OR = 3.06, 95% CI 1.93 to 4.84, *p* < 0.001), and self-report of falls (OR = 2.23, 95% CI 1.47 to 3.40, *p* < 0.001).

BMI and calf circumference were inversely associated with hip fractures (OR = 0.91, 95% CI 0.87 to 0.96, *p* < 0.001, and OR = 0.93, 95% CI 0.88–0.97, *p* = 0.006, respectively), while low gait speed increased the chance of hip fractures by almost two-fold (OR = 1.80, 95% CI 1.22 to 2.66, *p* = 0.003).

**Table 2:** Association of clinical characteristics, patient-reported outcomes, and muscle and aerobic capacity with the risk of hip fracture

Risk factors for hip fracture	Univariate OR (95% CI)	Multivariable Model^a^ OR (95% CI)	p
**Clinical characteristics**			
Multimorbidity^b^	1.15 (1.10–21.0)	1.12 (1.06–1.18)	< 0.001
Medications of long-term use	1.06 (1.00–1.12)	1.04 (0.98–1.10)	0.140
Number of falls	1.71 (1.57–1.87)	1.69 (1.52–1.80)	< 0.001
Sedative and/or tricyclic medications^c^	1.07 (0.63–1.66)	1.17 (0.75–1.82)	0.470
**Patient-reported outcomes**			
Regular or poor self-perception of health	1.51 (1.02–2.26)	1.59 (1.06–2.37)	0.022
Self-report of difficulty walking	4.69 (2.98–7.18)	3.06 (1.93–4.84)	< 0.001
Self-report of vision problems	1.60 (1.01–2.55)	1.29 (0.81–2.08)	0.270
Self-report falls	2.64 (1.74–4.00)	2.23 (1.47–3.40)	< 0.001
**Muscle and aerobic capacity**			
BMI^d^ (kg/m²)	0.88 (0.84–0.93)	0.91 (0.87–0.96)	< 0.001
Calf circumference	0.88 (0.84–0.93)	0.93 (0.88–0.97)	0.006
Slow gait speed^e^	2.94 (2.04–4.24)	1.80 (1.22–2.66)	0.003

^a^Adjusted for sex and age; ^b^number of medical diagnoses; ^c^alprazolam, bromazepam, clonazepam, diazepam, eszopiclone, phenobarbital, flunitrazepam, lorazepam, midazolam, nitrazepam, zolpidem, zopiclone, amitriptyline, nortriptyline, imipramine, clomipramine, maprotiline, and/or cyclobenzaprine; ^d^body mass index; ^e^gait speed > 11 s to cover 4 m

## Discussion

In this large retrospective cohort with real-world data of community-dwelling older adults, we found that clinical characteristics, aerobic and muscular capacity, and frailty-related PROs were associated with the risk of osteoporotic hip fracture, independently of age and sex. Namely, multimorbidity, the number of previous falls, regular or poor self-perception of health, self-report of walking difficulty, and self-report of falls increased the risk of hip osteoporotic fracture. In addition, calf circumference, gait speed, and BMI were inversely associated with the risk of hip fracture.

Multimorbidity was frequent in the study population and was associated with the risk of hip fracture. Indeed, multimorbidity is a prevalent problem in older adults, and a known risk factor for fractures in older adults [14, 15]. For instance, a cross-sectional study using data from a Japanese cohort study, which included 1420 adults over 60, showed that multimorbidity increased the risk of clinical frailty fractures independent of falls in the community-dwelling older adults’ population [14].

Interestingly, we did not find an association between fractures and sedative or tricyclic medications or long-term use medications in general. Antidepressants and sedative drugs are known risk factors for fractures [17], but the risk may vary among different medications and by dose and duration of use. This may explain why we found no association, as we assessed them as a large group instead of by subcategories or medications individually. The same is true for long-term medications, as this may include vitamins or supplements and may not accurately represent the real fracture risk.

Falls are a significant risk factor for proximal femoral fractures, as more than 90% of hip fractures may occur secondarily to falls [23]. We have found that either self-report falls (as a part of the IVCF-20 questionnaire) or fall registers in the EHR by health professionals were associated with increased fracture risk. Other PROs related to gait disturbances, like overall self-perception of health and self-report of walking difficulty, were also associated with fractures. PROs like the ones in the IVCF-20 questionnaire provide relevant information about the patient’s self-perception of health. They are easily obtained, therefore representing a promising tool for assessing risk fractures in the outpatient setting [10, 11]. Notably, the self-report of vision problems was not associated with fracture risk. The issue could be that the question about vision problems is too broad, so include patients with different vision loss categories in the same group.

Sarcopenia is a multidimensional syndrome closely related to falls and low bone mineral density [20, 21]. However, precise tools to assess sarcopenia are not widely available in clinical practice, and the health professional may appeal to surrogate parameters of muscle mass, strength, and performance. For instance, a cross-sectional study with 526 adults aged 40–89 years showed that calf circumference was positively correlated with appendicular skeletal muscle mass and skeletal muscle index [22]. We found that BMI and calf circumference were inversely associated with hip fracture, probably reflecting a higher muscle mass. In agreement with our findings, a systematic review and meta‑analysis of 35 studies with 1,5 million subjects showed that being an obese patient (BMI > 30 kg/m²) was associated with a decreased risk of hip fractures (pooled OR = 0.58, 95% CI 0.34–0.99) [24]. We also found that low gait speed increased the chance of hip fractures by almost two-fold, representing patients with poor physical performance and at risk of sarcopenia. Slow gait speed has been shown to predict hip fractures independent of bone mineral density [25]. This finding aligns with a study that showed that slow gait speed increased the risk of osteoporotic hip fractures in older men (HR 2.37, 95% CI 1.54–3.63) [26]. Besides that, and even more important is the fact that gait speed improves the predictive ability of FRAX [27].

The present study has limitations inherent to its observational design, such as a causal inference cannot be reliably established. In addition, several other residual confounders cannot be ruled out, despite adjustments for the most potential confounding variables like age and sex. Furthermore, socioeconomic adjustment, a characteristic associated with an increased risk of hip fracture [28], was not doable since this information is not routinely collected during medical appointments. Nevertheless, as all patients come from private health care and most live in the same city, they are expected to have a similar socioeconomic level. We also did not specify the medications used, which could provide a more detailed assessment of the risk factors for hip osteoporotic fracture. Besides, we cannot rule out the possibility that some patients followed at the clinic who suffered a hip fracture were not hospitalized, resulting in a loss of cases. Additionally, as a real-world evidence study where evidence is generated from data relating to patient health or experience or care delivery collected outside of highly controlled clinical trials [12], we had to use routine measures and instruments from clinical practice. Thus, one of the limitations was the use of BMI as a surrogate parameter of sarcopenia since anthropometry has been identified as a poor measure of muscle mass [29].. Another limitation was using only the IVCF-20 as a PROM. Furthermore, we could not access the patients’ X-rays, to confirm the discharge summary description.

Despite some limitations, our study includes the database of longitudinal health status and health care of a significant sample of individuals, generating real-world evidence, which may be especially useful for investigating the risk of osteoporotic hip fracture among community-dwelling older adults.

## Conclusion

Our study reinforces the importance of identifying risk factors for hip fracture in community-dwelling older adults beyond bone mineral density and available fracture risk assessment tools. Data obtained in primary care can help physicians, other health professionals, and public health policies to identify patients at increased risk of hip fractures.

## Data Availability

The datasets used and/or analyzed during the current study are available from the corresponding author on reasonable request.
